# Exploration of complex visual feature spaces for object perception

**DOI:** 10.3389/fncom.2014.00106

**Published:** 2014-09-12

**Authors:** Daniel D. Leeds, John A. Pyles, Michael J. Tarr

**Affiliations:** ^1^Computer and Information Science Department, Fordham UniversityBronx, NY, USA; ^2^Center for the Neural Basis of Cognition, Carnegie Mellon UniversityPittsburgh, PA, USA; ^3^Psychology Department, Carnegie Mellon UniversityPittsburgh, PA, USA

**Keywords:** neuroimaging, object recognition, computational modeling, intermediate feature representation, real-time stimulus selection

## Abstract

The mid- and high-level visual properties supporting object perception in the ventral visual pathway are poorly understood. In the absence of well-specified theory, many groups have adopted a data-driven approach in which they progressively interrogate neural units to establish each unit's selectivity. Such methods are challenging in that they require search through a wide space of feature models and stimuli using a limited number of samples. To more rapidly identify higher-level features underlying human cortical object perception, we implemented a novel functional magnetic resonance imaging method in which visual stimuli are selected in real-time based on BOLD responses to recently shown stimuli. This work was inspired by earlier primate physiology work, in which neural selectivity for mid-level features in IT was characterized using a simple parametric approach (Hung et al., 2012). To extend such work to human neuroimaging, we used natural and synthetic object stimuli embedded in feature spaces constructed on the basis of the complex visual properties of the objects themselves. During fMRI scanning, we employed a real-time search method to control continuous stimulus selection within each image space. This search was designed to maximize neural responses across a pre-determined 1 cm^3^ brain region within ventral cortex. To assess the value of this method for understanding object encoding, we examined both the behavior of the method itself and the complex visual properties the method identified as reliably activating selected brain regions. We observed: (1) Regions selective for both holistic and component object features and for a variety of surface properties; (2) Object stimulus pairs near one another in feature space that produce responses at the opposite extremes of the measured activity range. Together, these results suggest that real-time fMRI methods may yield more widely informative measures of selectivity within the broad classes of visual features associated with cortical object representation.

## 1. Introduction

Object recognition associates visual inputs—beginning with an array of light intensities falling on our retinas—with semantic categories, for example, “cow,” “car,” or “face.” Inspired by the architecture of the ventral occipito-temporal pathway of the human brain, models that attempt to implement or account for this process assume a feedforward architecture in which the features of representation progressively increase in complexity as information moves up the hierarchy (Riesenhuber and Poggio, 1999). The top layers of such a hierarchy are typically construed as high-level *object representations* that correspond to and allow the assignment of category-level labels. Critically, within such models, there is the presupposition of one or more levels of *intermediate* features that, while less complex than entire objects, nonetheless capture important—and compositional—object-level visual properties (Ullman et al., 2002). Yet, despite significant interest and study of biological vision, the nature of such putative intermediate features remains frustratingly elusive. To begin to address this gap, we explored the intermediate visual properties encoded within human visual cortex along the ventral pathway.

The majority of what we *have* learned about intermediate representation within the ventral cortex has come from primate neurophysiology studies. In a pioneering study, Tanaka (1996) explored the minimal visual stimulus sufficient to drive a given cortical neuron at a level equivalent to the complete object. He found that individual neurons in area TE were selective for a wide variety of simple patterns and that these patterns bore some resemblance to image features embedded within the objects initially used to elicit a response. Tanaka hypothesized that this pattern-specific selectivity has a columnar structure that maps out a high-dimensional feature space for representing visual objects. In more recent neurophysiological work, Yamane et al. (2008) and Hung et al. (2012) used a somewhat different search procedure to identify the contour selectivity of individual neurons in primate inferotemporal cortex (IT). Using a highly-constrained, parameterized stimulus space based on 2D contours, they found that most contour-selective neurons in IT encoded a subset of that parameter space. Importantly, each 2D contour within this space mapped to specific 3D surface properties—thus, collections of these contour-selective units should be sufficient to capture the 3D appearance of an object or part. At the same time, recent primate physiology and human fMRI studies have begun to address the issue of intermediate representations. For example, op de Beeck et al. (2001) and op de Beeck et al. (2008) demonstrated that the pattern of responses to complex synthetic stimuli in object-selective cortex is associated with perceived shape similarity and, in particular, that this intermediate region of visual cortex is sensitive to shape features such as curved vs. straight.

Also within the domain of human neuroscience, Kay et al. (2008) explored how responses—as measured by fMRI—of voxels coarsely coding for orientation and scale within human V1, V2, and V3 can be combined to reconstruct complex images. Although this work offers a demonstration of how human neuroimaging methods may support more fine-grained analyses (and inspiration for further investigation), it does not inform us regarding the nature of intermediate features. In particular, models of the featural properties of V1 and V2 are common, so Kay et al.'s study largely demonstrates that such models hold even at the voxel/millions-of-neurons level without explicating any new properties or principles for these visual areas. Put another way, Kay et al. decoded features within a well-understood parameter space in which it is already agreed that the particular brain regions in question encode information about the orientations and scales of local edges. In contrast, the core problem in identifying the features of intermediate-level object representation is that the parameter space is extremely large and highly underspecified, therefore it is difficult to find effective prior models that will fit the data. In this context, the proposal of Ullman et al. (2002) that intermediate features can be construed as image fragments of varying scale and location—leaving the contents of said fragments entirely unspecified—is still one of the strongest models of intermediate-level representation. In particular, this model predicts which task-relevant object information is likely to be encoded within the human ventral pathway (Harel et al., 2007).

Note that the large majority of models applied to biological object recognition have made weak assumptions regarding the nature of intermediate features (with the notable exception being Hummel and Biederman (1992) who made very strong assumptions as to the core features used in object representation; unfortunately, such strong assumptions worked against the generality of the model). For example, many models employ variants of Gabor filterbanks, detecting local edges in visual stimuli, to explain selectivities in primary visual cortex (V1) (Hubel and Wiesel, 1968). Extending this approach, both Kay et al. (2008) and Serre et al. (2007) propose hierarchies of linear and non-linear spatial pooling computations, with Gabor filters at the base, to model higher-level vision. In this vein, perhaps the most well-specified hierarchical model is “HMAX” (Cadieu et al., 2007) and its variants (Serre et al., 2007). While these models partially predict neural selectivity in the mid-level ventral stream (V4) for simple synthetic stimuli (Cadieu et al., 2007), HMAX imperfectly clusters images of real-world objects relative to the clusterings obtained from primate neurophysiology or human fMRI (Kriegeskorte et al., 2008).

To address the question of the intermediate-level features underlying neural object processing, we adopted two different models of visual representation. First, we explored a visual parameter space defined by “SIFT” (Lowe, 2004)—a method drawn from computer vision that we have, previously, established as effective in explaining some of the variance observed in the neural processing of objects (Leeds et al., 2013). Second, we explored a novel visual parameter space defined by collections of 3D components—akin to Biederman's approach (Hummel and Biederman, 1992)—“Fribble” objects (Williams and Simons, 2000). Both of these representational choices arise from a diverse set of linear and non-linear operations across image properties and, as such, can be thought of as proxies for more detailed models of visual representation within biological systems (see Leeds et al., 2013).

Using these two models, we collected fMRI data from human observers performing a simple object processing task using real-world objects characterized by coordinates in SIFT space or synthetic objects characterized by coordinates in Fribble space. That is, stimuli were projected onto one of two types of feature spaces, constructed to reflect the SIFT and Fribble models of object representation. During scanning, specific stimuli from these spaces were sequentially selected in *real-time* based on an algorithmic search of each feature space for images (and their corresponding image features) that produced *maximal* BOLD activity in a pre-selected brain region of interest (ROI) within the ventral visual pathway.

These novel methods allowed us to evaluate principles of object representation within human visual cortex. In particular, beyond the specifically-observed organizational structure of cortex, we found some evidence for “local inhibition,” in which cortical activity was reduced for viewing object images that varied slightly from preferred images for a given brain region. This finding expands on similar observations seen for earlier stages of visual processing (Hubel and Wiesel, 1968; Wang et al., 2012). With respect to topographic organization for objects, we observed that the object images producing the highest responses for a given ROI were often distributed across multiple areas of the visual feature space, potentially reflecting multiple neural populations with distinct selectivities encoded within small regions of visual cortex. Finally, across both real-world objects and Fribbles, we obtained some evidence for selectivity to local contours and textural surface properties.

Next we describe the novel methods that were integral to the execution of our study. In particular, we addressed two challenges. First, the potential space of object images, even given the reductions afforded by adopting SIFT or Fribble space, is massive. It was incumbent on us to implement a computationally-efficient image search strategy for stimulus selection. Second, because our goal was the real-time selection of stimuli, we developed a time-efficient means for measuring and processing BOLD signals.

## 2. Methods

### 2.1. Stimulus selection method

**Figure 1:**
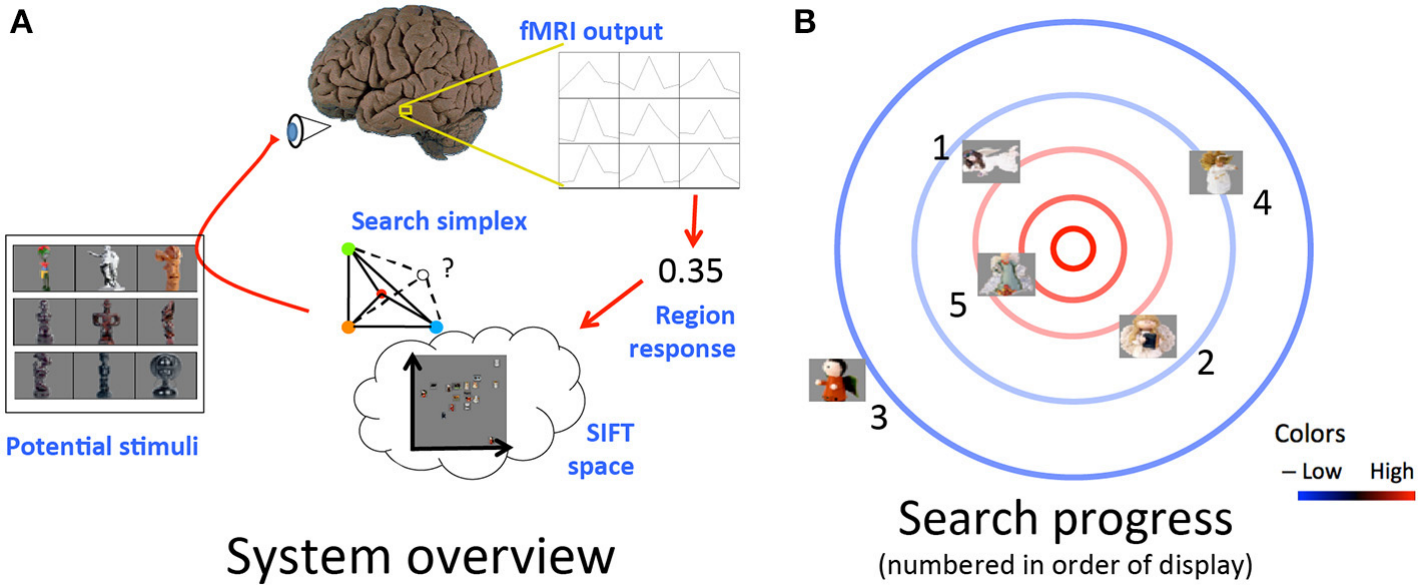
**(A)** Schematic of loop from stimulus display to measurement and extraction of cortical region response to selection of next stimulus. **(B)** Example progression of desired stimulus search. Cortical response is highest toward the center of the space (red contours) and lowest toward the edges of the space (blue contours). Stimuli displayed in order listed. Cortical responses to initial stimuli, e.g., those numbered 1, 2, and 3, influence selection of further stimuli closer to maximal response region in visual space, e.g., those numbered 4 and 5.

We developed methods for the dynamic selection of stimuli, choosing new images to display based on the BOLD response to previous images within a given pre-selected brain region. This search chooses each new stimulus by considering a space of visual properties and probing locations in this space (corresponding to stimuli with particular visual properties) in order to efficiently identify those locations that are likely to elicit maximal activity from the brain region under study (Figure 1). Each stimulus *i* that could be displayed is assigned a point in space *p*_*i*_ based on its visual properties. The measured response of the brain region to this stimulus *r*_*i*_ is understood as:

(1) $$r_{i}=f(p_{i})+\eta$$

That is, a function *f* of the stimulus' visual properties as encoded by its location in the representational space plus a noise term η, drawn from a zero-centered Gaussian distribution. The process of displaying an image, recording the ensuing cortical activity via fMRI, and isolating the response of the brain region of interest using the preprocessing program we model as performing an evaluation under noise of the function describing the region's response. For simplicity's sake, we perform stimulus selection assuming our chosen brain region has a selectivity function *f* that reaches a maximum at a certain point in the visual space and falls off with increasing Euclidean distance from this point. Under these assumptions, we use a modified version of the simplex simulated annealing Matlab code available from Donckels (2012), implementing the algorithm from Cardoso et al. (1996). An idealized example of what a search run might look like based on this algorithm is shown in Figure 1B. For each group, we performed searches in each of two scan sessions, starting at distinct points in the feature space for each session to probe the consistency of search results across different initial simplex settings. Further details are provided by Leeds (2013) and Cardoso et al. (1996).

### 2.2. Stimulus display

All stimuli were presented using MATLAB (2012) and the Psychophysics Toolbox (Brainard, 1997; Pelli, 1997) controlled by an Apple Macintosh and were displayed on a BOLD screen (Cambridge Research, Inc.) 24 inch MR compatible LCD display placed at the head end of the bore. Subjects viewed the images through a mirror attached to the head coil with object stimuli subtending a visual angle of approximately 8.3° × 8.3°.

### 2.3. fMRI procedures

Subjects were scanned using a 3 T Siemens Verio MRI scanner with a 32-channel head coil. Functional images were acquired with a *T*2^*^-weighted echo-planar imaging (EPI) pulse sequence (31 oblique axial slices, in-plane resolution 2 × 2 mm, 3 mm slice thickness, no gap, sequential descending acquisition, repetition time *TR* = 2000 ms, echo time *TE* = 29 ms, flip angle = 72°, GRAPPA = 2, matrix size = 96 × 96, field of view FOV = 192 mm). An MP-RAGE sequence (1 × 1 × 1 mm, 176 sagittal slices, *TR* = 1870, *TI* = 1100, *FA* = 8°, GRAPPA = 2) was used for anatomical imaging.

### 2.4. Experimental design

**Figure 2:**
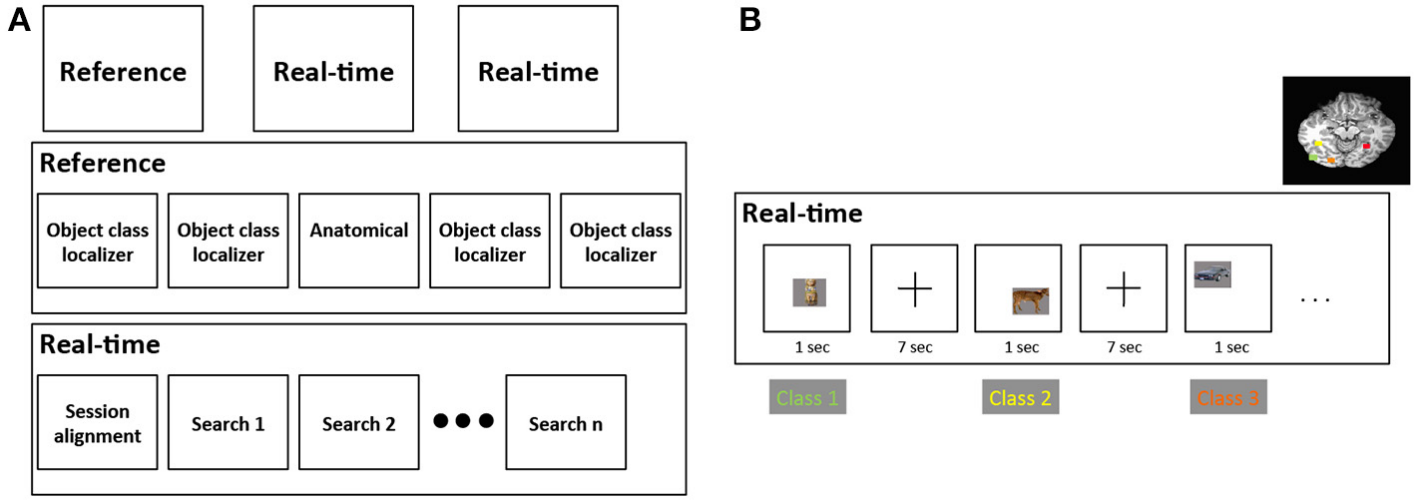
**(A)** Structure of the three scanning sessions performed for each subject. *First row* depicts the three sessions, *second row* depicts the runs for the reference session, and *third row* depicts the runs for each real-time session. **(B)** An example of the alternation among four stimulus class searches in a real-time search run. These four classes are comprised of mammals, human-forms, cars, and containers, and correspond to four colored brain regions shown on the upper-right of the figure.

For each subject, our study was divided into an initial “reference” scanning session and two “real-time” scanning sessions (Figure 2A). In the reference session we gathered cortical responses to four classes of object stimuli to identify cortical regions selective for each separate stimulus class. As described in Sections 2.6 and 2.7, two different stimulus sets, comprised of four visually-similar object classes, were used to explore visual feature selectivity: real-world objects and synthetic “Fribble” objects; each subject viewed stimuli from only one set. In the real-time scan sessions we searched for stimuli producing the maximal response from each of the four brain regions, dynamically choosing new stimuli based on each region's responses to recently shown stimuli.

Runs in the reference scan session followed a slow event-related design. Each stimulus was displayed in the center of the screen for 2 s followed by a blank 53% gray screen shown for a time period randomly selected to be between 500 and 3000 ms, followed by a centered fixation cross that remained displayed until the end of each 10 s trial, at which point the next trial began. As such, the SOA between consecutive stimulus displays was fixed at 10 s. Subjects were instructed to press a button when the fixation cross appeared. The fixation onset detection task was used to engage subject attention throughout the experiment. No other task was required of subjects, meaning that the scan assessed object perception under passive viewing conditions.

The stimuli were presented in four 3-min runs, spread across the 1-h scanning sessions and arranged to minimize potential adaptation and priming effects. Each run contained 36 object pictures, 9 objects from each of the four classes, ordered to alternate among the four classes. Stimulus order was randomized across runs. Over the course of the experiment, each subject viewed each picture four times; averaging across multiple repetitions was performed for each stimulus, described below, to reduce trial-by-trial noise. We determined from data gathered in Leeds et al. (2013) that relatively little information is gained by averaging over more than four repetitions.

To provide anatomical information, a T1-weighted structural MRI was performed between runs within the reference scan session.

In each of the two 1.5-h real-time scan sessions, the image selectivities of four distinct brain regions within ventral cortex were explored. For each brain region, a distinct search was performed using stimuli drawn from a single class of visual objects. Stimuli were presented for each search in 8.5-min “search” runs (4–8 runs were used per subject depending on other factors). Each stimulus was selected by the real-time search program based on responses of a pre-selected region of interest (ROI) to stimuli previously shown from the same object category. Each run contained 60 object pictures, 15 objects from each object class, ordered to alternate through the four classes—that is, search 1 → search 2 → search 3 → search 4 → search 1 …—as illustrated in Figure 2B. Alternation among distinct searches employing visually-distinct classes was advantageous in decreasing the risk of cortical adaptation that would have been present if multiple similar stimuli had been shown in direct succession. The focus of each search within an object class also limited visual variability across stimuli within that search. This also enabled the remaining sources of variability to be more intuitively identified and more readily associated with their influence on the magnitude of cortical activity. Note that the overt task during search runs varied depending on the stimuli shown. Task details are provided in Sections 2.6.4 and 2.7.4.

Each real-time session began with a 318-s functional scan performed with a viewing task to engage subject attention. The first functional volume scanned for this task was used to align the ROI masks (defined in Sections 2.6.5 and 2.7.5) selected from the reference session for a given subject to the subject's brain's position in the current session. This alignment corrects for changes in head position between the reference and the real-time scan sessions that might result in the brain, and its associated ROIs, moving to different locations in the scan volume. The remaining data volumes from this beginning task were ignored in that this task was designed simple to occupy the attention of the subject while computing inter-session brain alignment to be used for the remainder of the session.

### 2.5. Preprocessing

During analyses of the reference scan session, functional scans were coregistered to the anatomical image and motion corrected using AFNI (Pittman, 2011). Highpass filtering was implemented in AFNI by removing sinusoidal trends with periods of half and full length of each run (338 s) as well as polynomial trends of orders one through three. The data then were normalized so that each voxel's time course was zero-mean and unit variance (Just et al., 2010). To allow multivariate analysis to exploit information present at high spatial frequencies, no spatial smoothing was performed (Swisher et al., 2010).

During real-time scan sessions, functional volumes were motion corrected using AFNI. Polynomial trends of orders one through three were removed. The data then were normalized for each voxel by subtracting the average and dividing by the standard deviation, obtained from the currently analyzed response and from the previous “reference” scan session, respectively, to approximate zero-mean and unit variance (Just et al., 2010). The standard deviation was determined from 1 h of recorded signal from the reference scan session to gain a more reliable estimate of signal variability in each voxel. Due to variations in baseline signal magnitude across and within scans, each voxel's mean signal value required updating based on activity in each block (the time covering the responses for two consecutive trials). To allow multivariate analysis to exploit information present at high spatial frequencies, no spatial smoothing was performed (Swisher et al., 2010).

Matlab was used to perform further processing on the fMRI time courses for the voxels in the cortical region of interest for the associated search. For each stimulus presentation, the measured response of each voxel consisted of five data samples starting 2 s/1 TR after onset. Each five-sample response was consolidated into a weighted sum by computing the dot product of the response and the average hemodynamic response function (HRF) for the associated region. The HRF was determined based on data from the reference scan session. The pattern of voxel responses across the region was consolidated further into a single scalar response value by computing a similar weighted sum. Like the HRF, the voxel weights were determined from reference scan data. The weights corresponded to the most common multi-voxel pattern observed in the region during the earlier scan; that is, the first principal component of the set of multi-voxel patterns. This projection of recorded real-time responses onto the first principal component treats the activity across the region of interest as a single locally-distributed code, emphasizing voxels whose contributions to this code are most significant and de-emphasizing those voxels with typically weak contributions to the average pattern.

During the alignment run of each real-time session, AFNI was used to compute an alignment transformation between the initial functional volume of the localizer and the first functional volume recorded during the reference scan session. The transformation computed between the first real-time volume and the first reference volume was applied in reverse to each voxel in the four ROIs determined from the reference scan.

### 2.6. Real-world objects embedded in SIFT space

We pursued two methods to search for visual feature selectivity. In our first method, we focused on the perception of real-world objects with visual features represented by the scale invariant feature transform (SIFT, Lowe, 2004).

#### 2.6.1. Subjects

Ten subjects (four female, age range 19–31) from the Carnegie Mellon University community participated, provided written informed consent, and were monetarily compensated for their participation. All procedures were approved by the Institutional Review Board of Carnegie Mellon University.

#### 2.6.2. Stimuli

**Figure 3:**
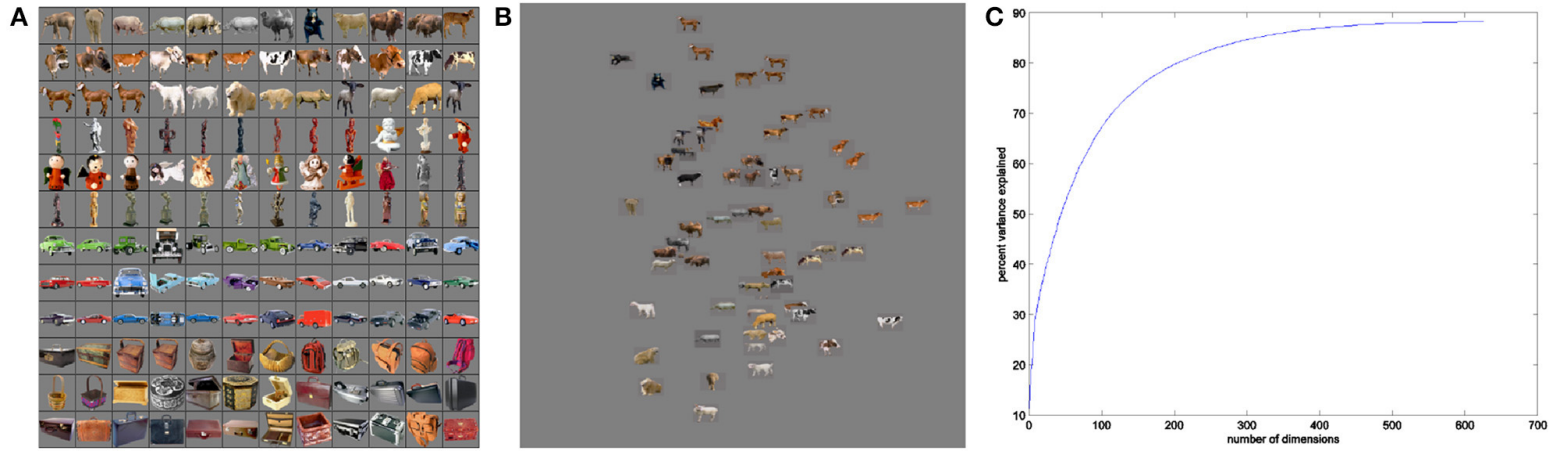
**Example real-world objects **(A)** and corresponding SIFT feature space **(B)****. Real-world object images were selected from four object classes—mammals, human-forms, cars, and containers. Feature space shows example stimuli projected onto first two dimensions of space. **(C)** Percent variance explained using first *n* dimensions of MDS feature space for SIFT.

Stimulus images were drawn from a picture set comprised of 400 distinct color object photos displayed on 53% gray backgrounds (Figure 3A). The photographic images were taken from the Hemera Photo Objects dataset from Hemera Technologies (2000–2003). The number of distinct exemplars in each object class varied from 68 to 150 object images. Note that our use of real-world images of objects rather than the hand-drawn or computer-generated stimuli employed in past studies of intermediate-level visual coding (e.g., Cadieu et al., 2007; Yamane et al., 2008) was intended to more accurately capture a broad set of naturally-occurring visual features.

#### 2.6.3. Defining SIFT space

Our real-world stimuli were organized into a Euclidean space (Figure 3B) that was constructed to reflect a scale invariant feature transform (SIFT) representation of object images (Lowe, 2004). Leeds et al. (2013) found that a SIFT-based representation of visual objects was the best match among several machine vision models in accounting for the neural encoding of objects in mid-level visual areas along the ventral visual pathway. The SIFT measure groups stimuli according to a distance matrix for object pairs (Leeds et al., 2013). In our present work, we defined a Euclidean space based on the distance matrix using Matlab's implementation of metric multidimensional scaling (MDS, Seber, 1984). MDS finds a space in which the original pairwise distances between data points—that is, SIFT distances between stimuli—are maximally preserved for any given *n* dimensions. This focus on maintaining the SIFT-defined visual similarity groupings among stimuli—using MDS—was motivated by the observations of Kriegeskorte et al. (2008) and Edelman and Shahbazi (2012), both of whom argued for the value of studying representational similarities to understand cortical vision.

The specific Euclidean space used in our study was derived from a SIFT-based distance matrix for 1600 Hemera photo objects, containing the 500 stimuli available for display across the real-time searches, as well as 1100 additional stimuli included to further capture visual diversity across the appearances of real-world objects (nb. ideally, the object space would be better covered by many more than 1600 objects, however, we necessarily had to restrict the total number of objects in order to limit the computation time required to generate large distance matrices). This distance matrix was computed using a “bag of words” method (Nowak et al., 2006; Leeds et al., 2013):

1. Several SIFT feature vectors were computed for each stimulus
2. 128 visual words were defined to describe typical feature vectors appearing in the 1600 photo objects
3. Each vector for each stimulus was assigned to its closest “word”
4. Each stimulus was represented by a histogram counting the number of times each word occurs in the image
5. Stimulus pairs were compared using the Kullback-Leibler divergence (Kullback and Leibler, 1951) between their corresponding histograms

MDS was then used to generate a Euclidean space into which all stimulus images were projected. The real-time searches for each object class operated within the same MDS space. This method produced an MDS space containing over 600 dimensions. Unfortunately, as the number of dimensions in a search space increases, the sparsity of data in the space will increase exponentially. As such, any conclusions regarding the underlying selectivity function will become increasingly more uncertain absent further search constraints. To address this challenge, we constrained our real-time searches to use only the four most-representative dimensions from the MDS space.

#### 2.6.4. Experimental design

Search runs in the real-time scan sessions employed a one-back location task to engage subject attention throughout the experiment. Each stimulus was displayed centered on one of nine locations on the screen for 1 s followed by a centered fixation cross that remained until the end of each 8 s trial, at which point the next trial began. Subjects were instructed to press a button when the image shown in this subsequent trial was centered on the same location as the image shown in the previous trial. The specific nine locations were defined by centering the stimulus at +2.5, 0, or −2.5° horizontally and/or vertically displaced from the screen center. From one trial to the next, the stimulus center shifted with a 30% probability.

#### 2.6.5. Selection of regions of interest (ROIs)

Reference scan data was used to select ROIs for further study in real-time scan sessions.

Class localizer: For each stimulus class *S*, selectivity *s*_*c*_ was assessed for each voxel by computing:

(2) $$s_{c}=\frac{\langle r_{c}\rangle -\langle r_{\bar{c}}\rangle }{\sigma (r_{c})}$$

where 〈*r*_*c*_〉 is the mean response for stimuli within the given class *c*, 〈*r*_*c*_〉 is the mean response for stimuli outside of the class *c*, and σ(*r*_*c*_) is the standard deviation of responses within the class^1^. We identified clusters of voxels with the highest relative responses for the given class using a manually-selected threshold and clustering through AFNI.

SIFT localizer: The representational dissimilarity matrix-searchlight method described in Leeds et al. (2013) was used to determine brain regions with neural representations of objects similar to the representation of the same objects by SIFT. Thresholds were adjusted by hand to find contiguous clusters with high voxel sphere *z* values.

Selection of ROIs: Visual inspection was used to find overlaps between the class-selective and SIFT-representational regions. For each class, a 125 voxel cube ROI was selected based on the observed overlap in a location in the ventral visual stream. The use of relatively small—one cubic centimeter—cortical regions enables exploration of local selectivities for complex visual properties. Analyses were successfully pursued on similar spatial scales in Leeds et al. (2013), using 123-voxel searchlights.

### 2.7. Fribble objects embedded in Fribble space

Our second attempt to search for visual feature selectivity focused on the perception of synthetic novel objects—Fribbles—in which visual features were parameterized as interchangeable 3D components (Williams and Simons, 2000).

#### 2.7.1. Subjects

Ten subjects (six female, age range 21–43) from the Carnegie Mellon University community participated, provided written informed consent, and were monitarily compensated for their participation. All procedures were approved by the Institutional Review Board of Carnegie Mellon University.

#### 2.7.2. Stimuli

**Figure 4:**
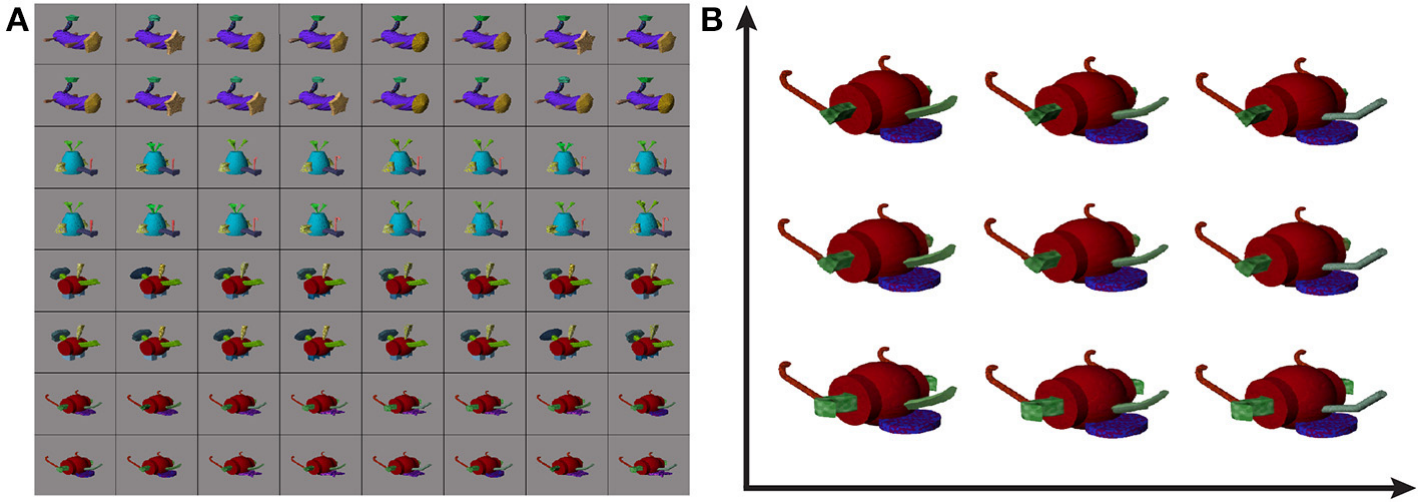
**Example Fribble objects (A) and example corresponding Fribble feature space (B)**. Fribble images were selected from four synthesized classes, shown in rows 1/2, 3/4, 5/6, and 7/8, respectively. Feature space shows stimuli projected onto first two dimensions of space.

Stimulus images were generated based on a library of synthetic Fribbles (Williams and Simons, 2000; Tarr, 2013), and were displayed on 54% gray backgrounds as in Section 2.6.2. Fribbles are animal-like objects composed of colored, textured geometric shapes. They are divided into classes, each defined by a specific body form and a set of four locations for attached appendages. In the library, each appendage has three potential shapes, e.g., a circle, star, or square head for the first class in Figure 4A, with potentially variable corresponding textures. These stimuli provide a careful control on the varying properties displayed to subjects, in contrast to the more natural, but less parameterized real-world objects.

#### 2.7.3. Defining Fribble space

We organized our Fribble stimuli into Euclidean spaces. In the space for a given Fribble class, movement along an axis corresponded to morphing the shape of an associated appendage. For example, for the purple-bodied Fribble class, the axes were assigned to (1) the tan head, (2) the green tail tip, and (3) the brown legs, with the legs grouped and morphed together as a single appendage type. Valid locations on each axis spanned from −1 to 1 representing two end-point shapes for the associated appendage, (e.g., a circle head or a star head). Appendage appearance at intermediate locations was computed through the morphing program Norrkross MorphX (Wennerberg, 2009) based on the two end-point shapes. Example morphs can be seen in the Fribble space visualization in Figure 4B.

For each Fribble class, stimuli were generated for each of 7 locations—the end-points −1 and 1 as well as coordinates −0.66, −0.33, 0, 0.33, and 0.66—on each of 3 axes, i.e., 7^3^ = 343 locations. A separate space was searched for each class of Fribble objects.

#### 2.7.4. Experimental design

Search runs in the real-time scan sessions employed a dimness detection task to engage subject attention throughout the experiment. Each stimulus was displayed in the center of the screen for 1 s followed by a centered fixation cross that remained displayed until the end of each 8 s trial, at which point the next trial began. On any trial there was a 10% chance the stimulus would be displayed as a darker version of itself—namely, the stimulus' red, green, and blue color values each would be decreased by 50 (max intensity 256). Subjects were instructed to press a button when the image appeared to be “dim or dark.” For the Fribble stimuli, the dimness detection task was used to address concerns we had regarding the one-back location task used with real-world object stimuli. In particular, the fact that subjects necessarily had to hold two objects in memory simultaneously in order to perform the one-back location task may have “blurred” our ability to assess the neural representation of single objects on any given trial. This confound may have limited the strength of real-world object search results. Thus, our change to the dimness detection task.

#### 2.7.5. Selection of Fribble class regions of interest

We employed the representational dissimilarity matrix-searchlight procedure of Leeds et al. (2013) to identify cortical areas whose visual representations are well characterized by each simple Fribble space. ROIs were selected manually from these areas for study during the real-time scan sessions. In these regions, we could search effectively for complex featural selectivities using the associated Fribble space.

## 3. Results

Our study was designed to explore complex visual properties utilized for object perception by the ventral pathway of the brain. We studied the distribution of recorded ROI responses in our novel visual feature spaces, defined and explored separately for real-world objects and for Fribble objects.

### 3.1. Visualizing feature spaces

To search for those visual properties selectively activating different cortical regions within the ventral pathway we constructed two types of visual feature spaces. Each of these spaces—Euclidean in nature—represented an array of complex visual properties through the spatial grouping of image stimuli that were considered similar according to the defining visual metric, as in Sections 2.6.3 and 2.7.3.

**Figure 5:**
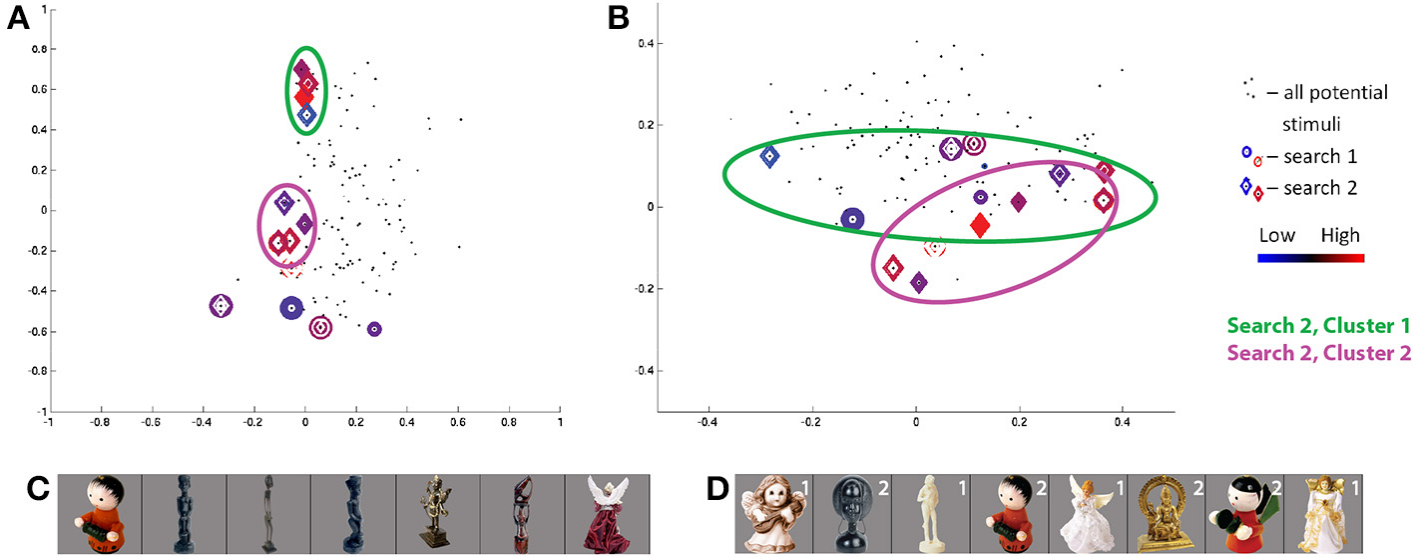
**Search results for S3, class 2 (human-forms), shown in (A) first and second SIFT space dimensions and (B) third and fourth dimensions**. Location of all potential stimuli in space shown as black dots. Results from real-time scan session 1 are circles, results from real-time scan session 2 are diamonds. For stimuli “visited” (i.e., selected by the search) three or more times, colors span dark blue–blue–red–dark red for low through high responses. First and second clusters of points visited in second search session are highlighted by green and pink circles, respectively. Note axes for **(A)** are from −1 to 1 and for **(B)** are from −0.5 to 0.5. **(C,D)** Stimuli visited three or more times in search session 1 **(C)** and search session 2 **(D)**, sorted in order of decreasing ROI response, averaged across all trials for each image. Stimuli from second search are labeled in white according to location in cluster 1 or 2.

Of note, each space contains a low number of dimensions—four dimensions for SIFT and three dimensions for each Fribble class—to allow the searches for visual selectivity to converge in the limited number of simplex steps that can be evaluated in real-time over the course of a scanning session. These low dimensional spaces also permit visualization of search activity over each scan session and visualization of general ROI response intensities across the continuum of visual properties represented by a given space. We display this information through colored scatter plots. For example, representing each stimulus as a point in feature space, Figure 5 shows the locations in SIFT-based space selected, or “visited,” by the search for human-form images evoking high activity in the pre-selected SIFT/“human-form” region of subject S3, and shows the regional response to each of the displayed stimuli. The four dimensions of SIFT-based space are projected onto its first two and second two dimensions in Figures 5A,B, respectively. Stimuli visited during the first and second real-time sessions are shown as circles and diamonds, respectively, centered at the stimuli's corresponding coordinates in the space. (Black dots correspond to the locations of all stimuli in the human-form class that were available for selection by the search program.) The magnitude of the average ROI response to a given visited stimulus is reflected in the color of its corresponding shape. For stimuli visited three or more times, colors span dark blue–blue–dark red–red for low through high average responses.

### 3.2. Real-time search behavior

In real-time scanning sessions, dynamic stimulus selection was pursued to more effectively explore each space of visual properties in limited scan time and to quickly identify visual properties producing the strongest activity from cortical regions in the ventral object perception pathway. Because the methods for real-time search are novel, we assess and confirm their expected performance in addition to studying the visual properties discovered by these methods. In particular, we expected each search in visual feature space to show the following two properties:

1. Convergence onto one, or a few, location(s) in the associated visual space producing greatest cortical response, corresponding to the regional selectivity.
2. Consistency in stimuli found to be preferred by the ROI, despite differing search starting points in visual feature space in the two scanning sessions for each subject.

However, because of the novelty of our methods—and thus our limited knowledge about optimal search parameters—and because of the limited number of stimulus display trials available, convergence occurred for only 10% of searches of real-world objects and 25% of searches of Fribble objects, as judged by a measure of convergence significance devised for our study (Section S1). We focus our ensuing analyses on the convergent and consistent searches. We anticipate further methodological development stemming from our present study will improve search convergence in future studies.

### 3.3. Selection of brain regions of interest

**Figure 6:**
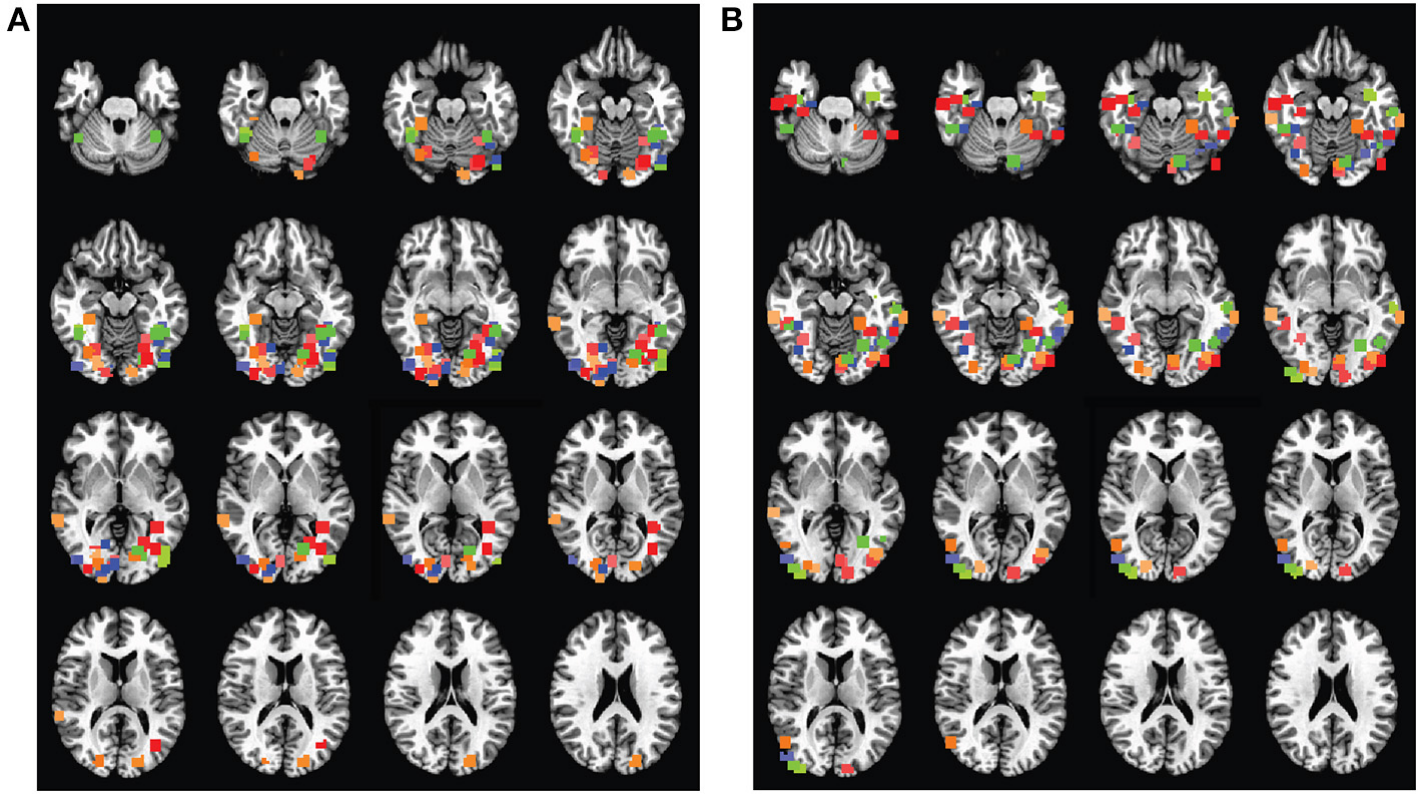
**Class-selective regions for 10 subjects in (A) real-world objects search and (B) Fribble objects search, projected onto the Talairach brain**. Colors are associated as follows (listed as real-world/Fribble, respectively): blue for mammals/purple curved tube object, green for human-forms/blue-bodied–yellow-winged object, orange for cars/bipedal–metal-tipped-tail object, red for containers/wheelbarrow object, overlaps shown as single color. Each subject is assigned a shade of each of the four colors.

Both for subjects viewing real-world objects and subjects viewing Fribble objects, ROIs containing cubes of 125 voxels were manually selected for each of four stimulus classes searched (Figure 6). Beyond incorporating voxels most highlighted by reference scan analyses reviewed above, the four regions for each subject were selected to be non-overlapping and to lie within the ventral pathway, with a preference for more anterior voxels, presumably involved in higher levels of object perception. With this selection approach in mind, consideration of the anatomical locations of the chosen ROIs provides perspective on the span of areas using SIFT-like and “Fribble-morph-like” representational structures across subjects, and the distribution of areas most strongly encoding each of the four studied object classes across subjects. We also gain perspective on the range of brain areas across subjects and searches studied for complex visual selectivities.

ROIs used for real-world object searches are distributed around and adjacent to the fusiform cortex, while ROIs used for Fribble object searches are distributed more broadly across the ventral pathway.

### 3.4. Complex visual selectivities

We examine cortical responses observed for stimuli displayed in searches, selected for convergence and consistency, to determine visual properties significant to ROI representations of visual objects. In particular, we study the frequently-visited stimuli, ranked by ROI responses, to intuit important visual properties for each ROI and use the scatter plot introduced in Section 3.1 to visualize cortical activity across visual space, as well as to observe search behavior. The adaptive trajectory of each real-time search further reflects ROI selectivities. In the following two sections, we use the feature space for real world objects and then the feature spaces for Fribble objects as powerful new tools for characterizing and understanding cortical responses to complex visual properties.

#### 3.4.1. Real-world objects search

Examination of points frequently visited by each search and the responses of the corresponding brain regions revealed (1) multiple distinct selectivities within search of single ROIs, (2) marked change in cortical response resulting from slight deviations in visual properties/slight changes in location in visual space, and (3) several intuitive classes of visual properties used by the ventral pathway—including surface texture as well as two- and three-dimensional shape.

We examine the results of the two “most-converged” searches in detail below, and summarize results for all other searches with above-threshold convergence.

The class 2/human-forms search in the second session for subject S3 was one of the most convergent. Projecting the visited stimuli along the first two dimensions in SIFT-based space in Figure 5A, and focusing on frequently-visited stimuli, we see two clusters, circled in green and pink. The images visually are split into two groups^2^ : one group containing light/generally-narrow-shapes and the second group containing less-light/wide-shapes, as shown in Figure 5D. Notably, stimuli evoking high and low responses appear in both clusters, and similar-looking images can elicit opposite ROI activities—e.g., the two red characters. We consider this as potential evidence of local inhibition.

The class 2 search in the first session for S3 shows a quite weak convergence measure. Unlike results for the second session, there is no concentration of focus around one (or two) spatial locations. Despite a very low consistency measure, there is evidence for a degree of consistency between session results. The stimuli evoking the strongest and weakest responses in the first session appear in the lower cluster of visited points in the second session. The red wingless character, again, elicits high response while the purple winged character in the first session and the red-green winged character in the second session, nearby in visual SIFT-based space, elicit low responses. The winged character in the first session is projected as a very small blue circle at (−0.05, 0.02, 0.15, 0.10) in the SIFT space in Figures 5A,B. By starting from a separate location, the second search finds two ROI response maxima in SIFT space.

**Figure 7:**
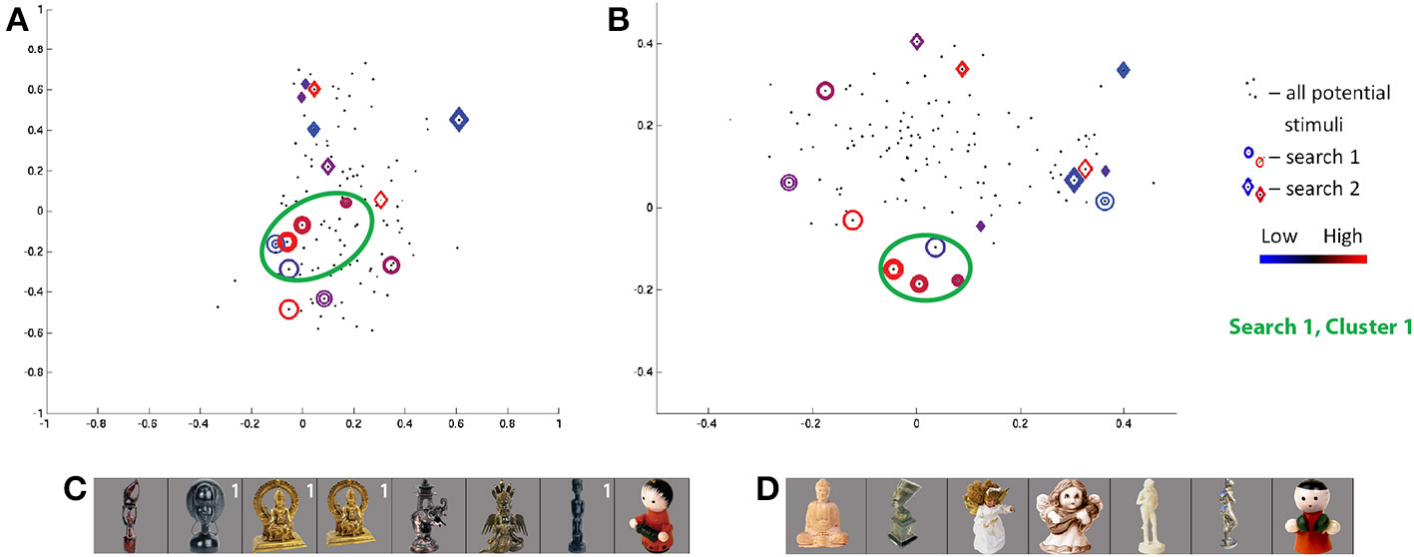
**Search results for S6, class 2 (human-forms), shown in (A) first and second SIFT space dimensions and (B) third and fourth dimensions**. Colors and shapes used as in Figure 5. The stimuli visited three or more times in the first **(C)** and second **(D)** search sessions are shown sorted in order of decreasing ROI response, averaged across all trials for each image. Stimuli from first search are labeled in white if located in cluster 1.

The class 2 search in the first session for S6 showed the greatest convergence measure across all searches. Projecting the visited stimuli along the SIFT dimensions in Figure 7, we see one cluster (of red and blue circles) around the coordinates (−0.1, −0.15, 0, −0.15) and several outliers for the first session. The three stimuli in the cluster producing the highest responses (Figure 7C) may be linked by their wide circular head/halo, while the smallest-response stimulus is notably thin—potentially indicating response intensity as a wide/thin indicator. Notably, stimuli evoking high and low responses, coming from the two ends of the wide/thin spectrum, are nearby one another in the part of the SIFT space under study by the search—a potential example of the limits of four SIFT dimensions to capture magnitudes of all visual differences among real-world objects.

The class 2 search in the second session for S6 shows a quite weak convergence measure. Similarly, as the consistency measure is low, the stimuli frequently visited in the second session fail to overlap with similar feature space locations and “similar-looking”^3^ stimuli frequently visited in the first session. Although a red character produces the minimum responses in each of the two searches (Figure 7), the two characters are located in distinct corners of the SIFT space (dark red diamond and blue circle in Figure 7).

Comparison of searches for S3 and S6, in Figures 5, 7, respectively, shows a similar pattern of visited stimuli in the feature space. For both subjects, there is a focus close to the first dimensional axis, i.e., a vertical line of red and blue circles and diamonds along the first two dimensions; visited stimuli follow a V pattern in the second two dimensions. Furthermore, some of the highest ROI response stimuli appear (in red) at high locations along the second and third dimensions. Similarly, frequently-visited stimuli for S6 session 1 (dark blue circles) appear close to the the observed lower cluster for S3 session 2, though the cortical responses for the two subjects appear to differ. Comparing Figures 5, 7, we also can confirm a degree of overlap between the images frequently shown for each subject. In both subjects, frequently visited stimuli seemed to show regional selectivity, and potentially differentiation, for narrow-versus-wide shapes.

Study of frequently visited stimuli in search sessions showing lower degrees of convergence reveals a mix of results, summarized in Table 1. Most searches identify one potential cluster producing marked high, and possibly marked low, responses from the ROI. A variety of visual properties are identified for different regions under study, from surface details to shapes of object parts. In one of the searches considered in the table, for subject S6, we note stimuli producing high and low cortical responses are close together in visual space.

**Table 1:** **Summary of results for additional convergent and consistent searches**.

**Subject/Class/Session**	**Local inhibition**	**# Cluster centers**	**Visual properties**
S1/2/1	No	1, many outliers	Metallic surfaces, rectangular base
			(uncertain)
S5/2/2	No	1, many outliers	Sharp local angles defining internal holes or feathers
S7/2/1	No	1	High spatial frequencies on surface, shiny spots
S7/2/2	No	1	(uncertain)
S1/4/1	No	1	(uncertain)
S6/4/1	No	2	Cluster 1: same object in different colors;
			Cluster 2: multiple long edges (uncertain)
S6/4/2	Yes	1	Top handles, horizontal and vertical lines (uncertain)

*“Local inhibition” marks the observation that stimuli close to one another in visual space evoke particularly high and low ROI responses. “(uncertain)” notes uncertainty about visual properties of frequently-visited stimuli clustered in feature space*.

**Figure 8:**
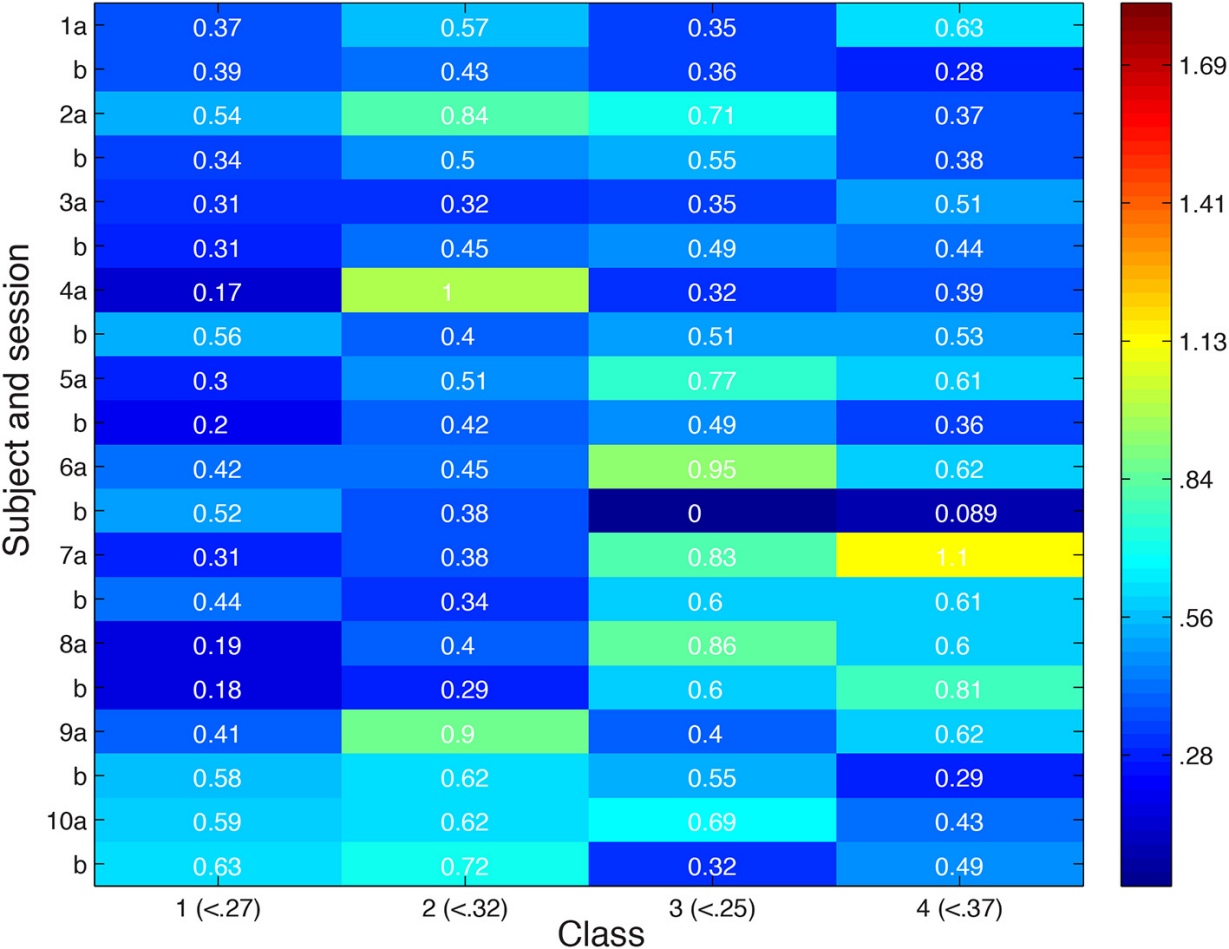
**Distance in feature space between stimulus pair evoking greatest difference in cortical responses or second greatest difference in cortical responses**. Feature space distance color coded from red (high) to blue (low). For each class, number in parentheses indicates distance one standard deviation below average distance between randomly-selected stimuli. Search session 1 is represented by letter “a” and session 2 by letter “b.”

Looking more broadly for evidence of local inhibition across both convergent and non-convergent searches, we measure the distance in feature space between stimuli producing the highest and lowest ROI responses, and compare it with the typical distribution of inter-stimulus distances in feature space in Figure 8. Stimuli were deemed to be close in space if their distance was more than a standard deviation below the average inter-stimulus pairwise distance among the stimuli in the class. Out of 80 searches performed, we observed nine in which nearby stimuli produced extremely different cortical responses.

A comparison of class 2 searches for S1, S3, and S6 reveals a similar pattern of stimulus responses in feature space. Qualitatively, the stimuli are arranged roughly linearly along the first two dimensions and show a more complex “V” pattern in the second two dimensions. Some of the highest ROI response stimuli appear at high locations along the second and third dimensions for S1 session 2, S3 session 2, and S6 session 2. Notably, the 4 human figures with largely-uniform white surfaces (Figure 5D) constituting the first cluster for S2 from session 2, were also frequently displayed to S1 in session 2; 3 of the 4 figures are sorted in the same order based on ROI response size.

In contrast, comparison of class 2 searches for S5 with those of the subjects reported above, S1, S3, and S6, shows a great degree of difference in the pattern of frequently visited stimuli in feature space and in the pattern of cortical responses across space. This finding reflects the expected diversity of selectivities employed in perception of a given object class, e.g., human-forms.

#### 3.4.2. Fribble objects search

Among subjects viewing Fribble objects, 20 selectivity searches converged and 7 searches showed consistency across search sessions. As in real-world object searches, examination of stimuli frequently visited by each search and the responses of the corresponding brain regions revealed (1) multiple distinct selectivities within search of single ROIs, (2) marked change in cortical response resulting from slight deviations in visual properties/slight changes in location in visual space, and (3) several perception approaches used by the ventral pathway—including focus on the form of one or multiple component “appendages” for a given Fribble object.

We examine in detail the results of two of the most convergent searches as well as the results of the two most inter-session consistent searches. We also summarize results for all other searches with above-threshold convergence and consistency.

**Figure 9:**
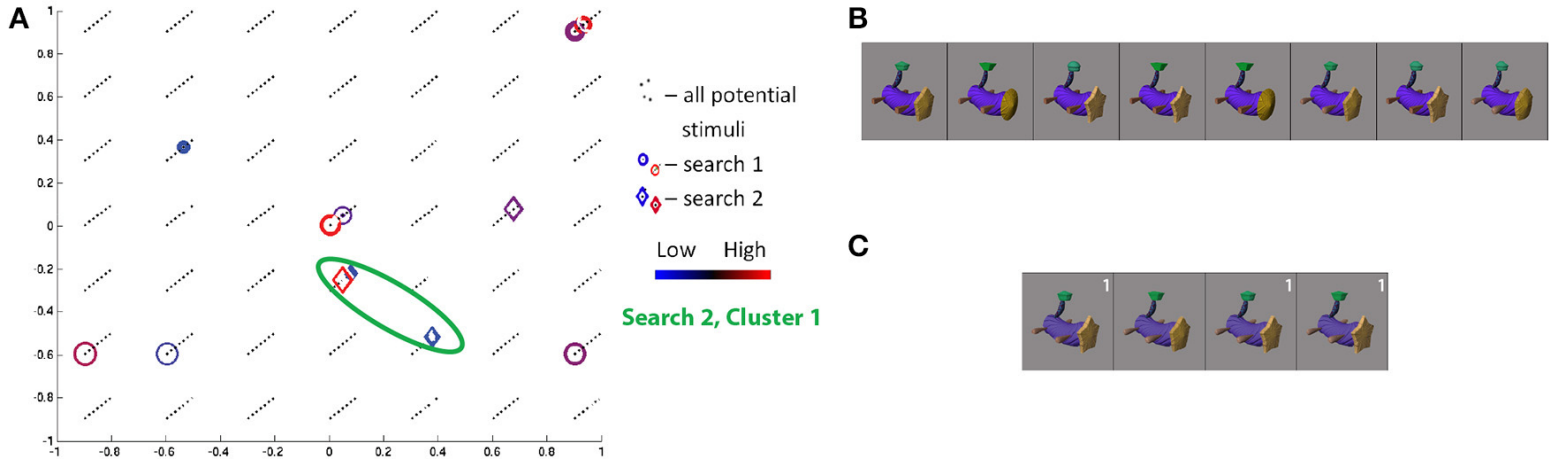
**Search results for S11, class 1, shown in three-dimensional Fribble space (A), with third dimension represented as diagonal offset**. Positive third dimension results in displacement up and to the right. Location of all potential stimuli in space shown as black dots. Results from real-time scan session 1 are circles, results from real-time scan session 2 are diamonds. For stimuli visited three or more times, colors span dark blue–blue–red–dark red for low through high responses. Cluster of points visited in second search session is highlighted by green circle. The stimuli visited three or more times in the first **(B)** and second **(C)** search sessions are shown sorted in order of decreasing ROI response, averaged across all trials for each image. Stimuli from second search is labeled white if located in cluster 1.

The class 1/curved tube object search in the second session for S11 showed high convergence. Projecting the visited stimuli along the three Fribble-specific morph dimensions in Figure 9A, noting the third dimension is indicated by diagonal displacement, we see one cluster^4^ (of red and blue diamonds) centered around (0, −0.33, 0.66). The cluster contains three of the four stimuli visited three or more times in the second session, as shown in Figure 9C. These stimuli show similar appearances in their three appendages. The outlying stimulus, while deviating in its more circular head and more flat-topped tail tip, retains the round leg shape of the three in-cluster stimuli. We observe Fribble ROIs sometimes are most selective for the shape of a subset of the component appendages, although clustering appears to indicate the head and tail-tip shape remain important for S11's ROI as well, as does cross-session comparison of results below.

The class 1 search in the first session for S11 shows quite weak convergence. Projecting the visited stimuli along the three Fribble-space dimensions (red and blue circles) shows the search spreading across the space. In several locations, pairs of near-adjacent stimuli were visited, as in the lower left, upper right, and center of Figure 9A. In each location, the stimuli evoked opposite strength responses from the ROI—the second and seventh highest responses are coupled, as are the first and sixth, and the third and seventh. Sensitivity to slight changes in visual features—potential local inhibition—thus is seen both for Fribble and real-world object perception.

**Figure 10:**
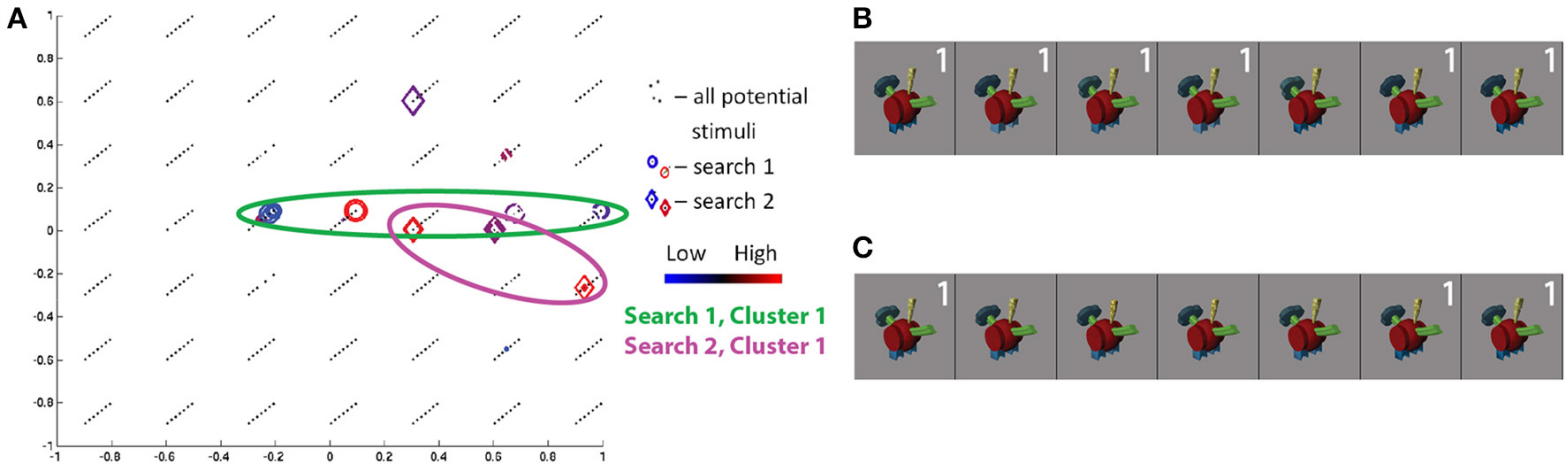
**Search results for S17, class 3, shown in three-dimensional Fribble space (A)**. Colors and shapes used as in Figure 9. The stimuli visited three or more times in the first **(B)** and second **(C)** search sessions are shown sorted in order of decreasing ROI response, averaged across all trials for each image. Stimuli from each search are labeled in white if located in cluster 1 for their respective searches.

The class 3/bipedal, metal-tipped tail object searches for S17 showed high cross-session consistency. Projecting the visited stimuli along the three Fribble-specific morph dimensions in Figure 10A, we see the first session focuses on the axis of dimension 1, the second session focuses on the axis of dimension 2, and both emphasize stimuli with *dim*2 ≈ 0. The visited points for each session spread widely, albeit roughly confined to a single axis. Visually, in Figures 10B,C, these stimuli are grouped for their spiked feet (*dim*2 = 0), as well as for their tails appearing half-way between a circle and a cog shape (see Figure 4A) and their yellow “plumes” half-way between a round, patterned and angled, uniformly-shaded shape. The importance of spike-shaped feet indicated in both searches, even beyond the (0, 0, 0.66) cluster focus, may relate to prominance of edge detection in biological vision, expanding to the detection of sharp angles. As seen for other Fribble and real-world objects searches above, stimuli evoking the lowest and highest responses are notably clustered in the search space.

Visual comparison of searches and of regional responses for different subjects cannot be made across classes, as each Fribble space is defined by a different set of morph operations. Within class comparisons do not reveal strong consistent patterns across ROIs.

**Figure 11:**
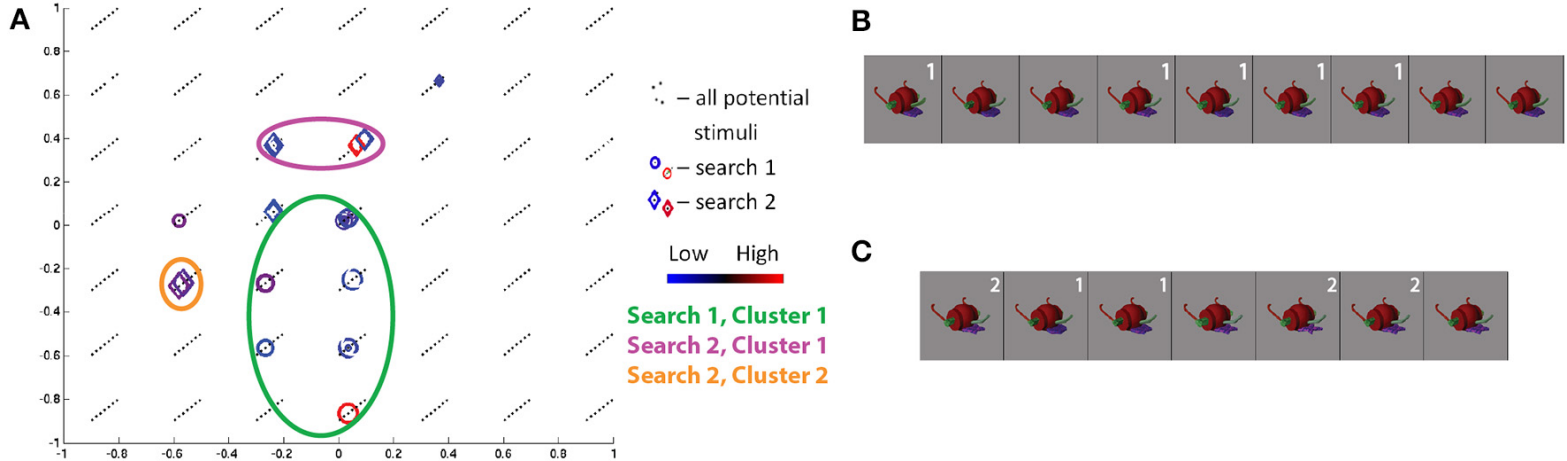
**Search results for S19, class 4, shown in three-dimensional Fribble space**. Colors and shapes used as in Figure 9. The stimuli visited three or more times in the first **(B)** and second **(C)** search sessions are shown sorted in order of decreasing ROI response, averaged across all trials for each image. Stimuli from each search are labeled in white if located in a cluster for their respective search.

The class 4/wheelbarrow object search for S19 showed high convergence in both sessions. Furthermore, the two searches together showed the highest cross-session consistency across all subjects and object classes. Projecting the visited stimuli along the three Fribble-specific morph dimensions in Figure 11A, we see clustering along *dim*1 = 0 and *dim*3 = −0.33 for the first session (red and blue circles); dimension 2 location of the stimuli is more broadly-distributed, but limited to *dim*2 ≤ 0. The stimuli at the center of the first session cluster, shown in Figure 11B, are linked by their purple tongue and green ear shapes. The ROI appears to be selective for the shape of a subset of component appendages, without regard for other elements of the object (i.e., the green nose). As observed throughout our search results, stimuli evoking high and low responses appear in the same cluster, sometimes adjacent to one another in space and appearing rather similar by visual inspection, indicating ROI sensitivity to slight changes in appearance.

Projecting the visited stimuli for the second session along the three Fribble dimensions (as red and blue diamonds) shows two clusters. The presence of multiple selectivity centers is consistent with observed ROI response properties for subjects viewing real-world objects, as well as Fribble subject S11 discussed above. The stimuli at the center of the larger second session cluster show a similar green ear and similar mid-extremes nose but a more star-shaped purple tongue. The two stimuli with the most-circular tongues form the second cluster. This second cluster has the highest consistency with two of the cluster outliers from the first session, i.e., the second and third most active stimuli for the first session. Stimuli evoking high and low responses appear in the same cluster, sometimes adjacent to one another in space and appearing rather similar by visual inspection.

Study of frequently visited stimuli in search sessions showing lower degrees of convergence reveals a mix of results, summarized in Table 2. Most searches identify one potential cluster producing marked high and low responses from the ROI. Most searches also show ROI selectivity for shapes of all three object appendages, each corresponding to a feature space dimension, though several searches indicate selectivity for only two appendages. In almost all the searches considered in the table, we note stimuli producing high and low cortical responses are close together in visual space.

**Table 2:** **Summary of results for additional convergent and consistent searches**.

**Subject/Class/Session**	**Local inhibition**	**# Cluster centers**	**# Selectivity dims**
S13/1/2	Yes	1	3
S16/1/2	Yes	1	3
S17/1/2	Yes	2	3
S16/2/1	Yes	1	3
S17/2/2	No	1	3
S16/3/1	Yes	2	2, 3
S18/3/1	Yes	1	2
S11/4/1	Yes	1	3
S18/4/1	Yes	1	3
S20/4/1	Yes	1	3

*“Local inhibition” marks the observation that stimuli close to one another in visual space evoke particularly high and low ROI repsonses. “# selectivity dims” indicates whether clustering occurs in all dimensions (entry is 3) or only along a few dimension (entry can be 1 or 2)*.

**Figure 12:**
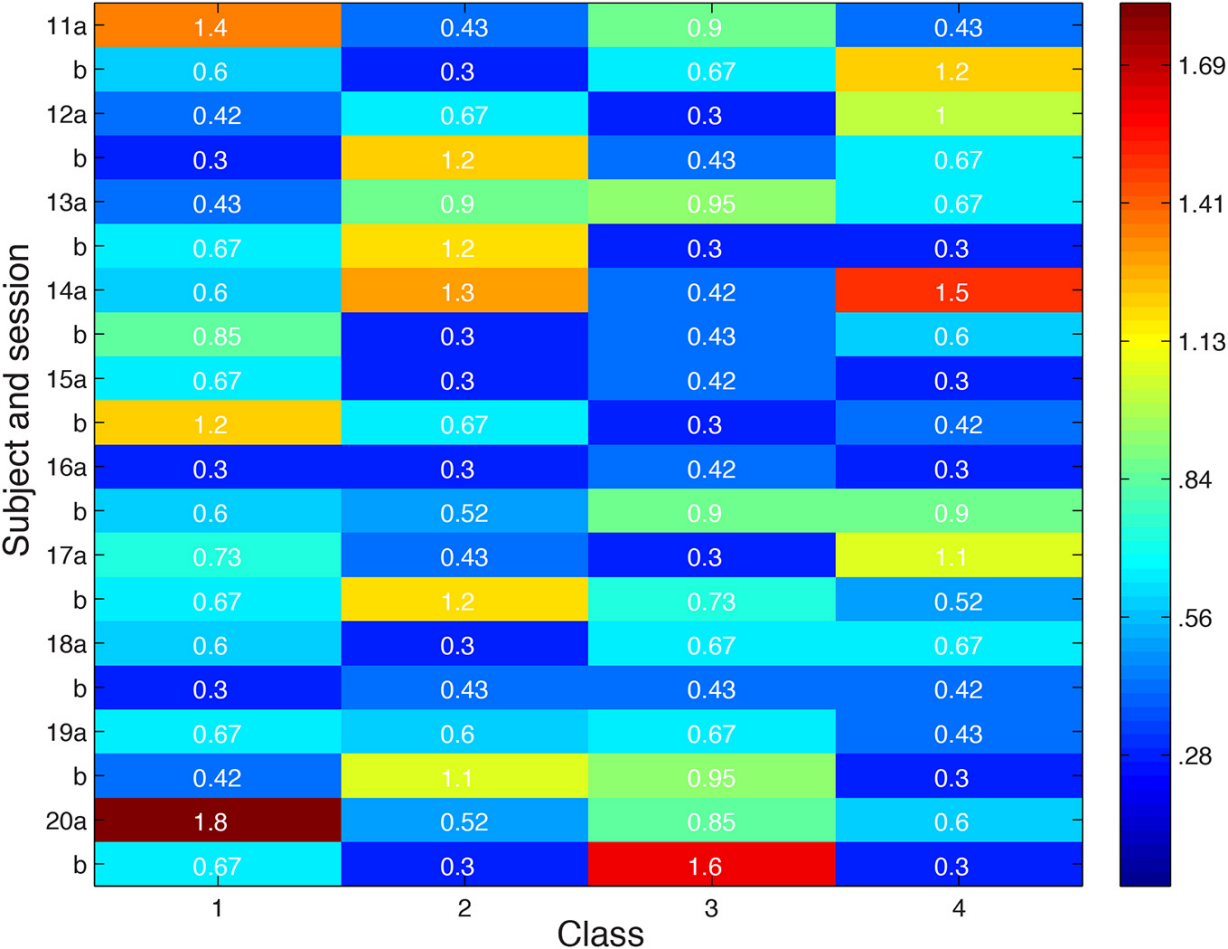
**Distance in feature space between stimulus pair evoking greatest difference in cortical responses or second greatest difference in cortical responses**. Feature space distance color coded from red to blue. Distance one standard deviation below average distance between randomly-selected stimuli is 0.87. Search session 1 is represented by letter “a” and session 2 by letter “b.”

Looking more broadly for evidence of local inhibition across both convergent and non-convergent searches, we measure the distance in feature space between stimuli producing the highest and lowest ROI responses, and compare it with the typical distribution of inter-stimulus distances in feature space in Figure 12. Stimuli were deemed to be close in space if their distance was less than 0.87. Notably, the minimum distance between a pair of stimulus points was 0.3. Out of eighty searches performed, we observed over 75% of searches in which nearby stimuli produced extremely different cortical responses. 50% of searches showed extremely different cortical responses for stimuli at most two minimum edit steps away in visual space, stepping between neighboring black dots in the scatter plots shown above.

In sum, searches in most ROIs discussed above cluster around a single location, indicating a single selectivity in visual space specific for all three component appendages in a given Fribble, though several searches find multiple clusters and some results show Fribble location along certain dimensions does not affect ROI response. Locations of clusters, and of high ROI responses, are roughly equally likely to be in the middle of the space (morphing between clear end-point shapes) or close to the extreme ends (showing clear end-point shapes like star heads or sharp-toed feet). For several (but not all) ROIs, stimuli close to one another in visual space evoked high and low cortical responses—indicating sensitivity to slight changes in visual properties.

### 3.5. Limitations of using SIFT multi-dimensional scaling space

The use of a SIFT-based Euclidean space yielded relatively poor search performance across subjects and ROIs, despite the abilities of SIFT to capture representations of groups of visual objects in cortical regions associated with “intermediate-level” visual processing, discussed by Leeds et al. (2013). Significant convergence and consistency was observed more rarely than expected—certainly compared to those statistics in Fribble spaces—and visual inspection of frequently-visited stimuli frequently failed to provide intuition about visual properties of importance to the brain region under study.

Confining the SIFT representation to four dimensions, found through multi-dimensional scaling as discussed in Section 2.6.3, limited SIFT space's descriptive power over the broad span of visual properties encompassed by real-world objects. Use of a small number of dimensions was required to enable effective search over a limited number of scan trials. However, Figure 3C shows that at least 50 dimensions would be required to explain 50% of the variance in a SIFT-based pairwise distance matrix for 1600 images. Even among the 100 stimuli employed for each object class, the four dimensions used account for less than 50% of variance. The missing dimensions account for grouping pairwise distance patterns across large sets of images—therefore, more-careful selection of stimuli included in a given object class still renders four-dimensional SIFT space insufficiently-descriptive.

Intuitively, it is not surprising that there are more than four axes required to describe the visual world, even in the non-linear pooling space of SIFT. Indeed, the method employed in our present study employs 128 descriptors and 128 visual words (Leeds et al., 2013). Further study shows that tailoring SIFT space for each of the four object classes used in our sessions still requires over 10 dimensions each to account for 50% of variance. The exploration of selectivities for real-world objects using Euclidean space may well require more dimensions, and thus more trials or a more efficient real-time analysis approach. The number of dimensions may be kept small by identification of a better-fitted feature set, or by limitations on the stimuli. We pursue the latter through Fribble spaces, with notable improvement.

For the real-world object searches, our use of multi-dimensional scaling to define SIFT space may also have obscured observations of unifying properties for the stimuli producing high cortical activation. In particular, MDS identifies dimensions maximizing the preservation of pairwise distances between images. Within the first few dimensions, MDS allows groups of objects deemed similar within SIFT to be clustered together—such clustering of visually-similar objects is one of the key assumptions we rely on in our stimulus selection methods. However, this representation of the stimulus images may not capture more subtle variations *within* a cluster of visually-similar objects. For example, within the mammal class, dogs may form a cluster clearly distinct from cows, but this method does not guarantee that two sitting dogs will be closer together within the dog cluster than a sitting dog and a standing dog. Similarly, we would not expect dogs to be sorted according to ear length (or many other intuitive properties) along any vector in SIFT space, even though we would expect all dogs to be spatially far from rhinoceroses. In contrast, Fribble space is defined to better capture such nuanced and graded visual variability, and, perhaps as a consequence, reveals ROIs invariant to changes in some dimensions but selective to changes in others.

Looking forward, we note that there exist a wide range of alternative feature spaces that might be explored in future studies. For example, real-world objects might be rated on a large number of visual properties (or properties could be automatically extracted using unsupervised statistical learning over a large number of images, e.g., Chen et al., 2013), and PCA could then be used to determine a smaller number of dimensions capturing common patterns among these properties—an approach that is somewhat of a compromise between SIFT space and Fribble space. At the same time, acknowledging the limitations of the SIFT-based space, we feel that our experimental findings provide some insight into visual selectivity within selected cortical regions across multiple subjects.

## 4. Discussion

Our overall goal was to better elucidate the complex visual properties used in visual object perception. In contrast to the field's understanding of early visual processing (e.g., edge detection in primary visual cortex), the intermediate-level visual features encoded in the ventral pathway are poorly understood. To address this gap, we relied on computational models of vision to build low-dimensional feature spaces as a framework in which to characterize neural activity across the high-dimensional world of visual objects. Whereas Hubel and Wiesel explored varying orientations and locations of edges to examine single neuron selectivity in primate V1 (Hubel and Wiesel, 1968), we explored these visual feature spaces to examine neural selectivity across 10 mm^3^ brain regions in the human ventral pathway.

Uniquely, we employed real-time fMRI to determine neural selectivity—rapidly identifying those visual properties that evoked maximal responses within brain regions of interest in the context of limited scanning time. These real-time searches across visual feature space(s) provide new understanding of the complex visual properties encoded in mid- and high-level brain regions in the ventral pathway. First, we found that individual brain regions produced high responses for several sets of visual properties, that is, for two or three locations within a given feature space. Second, we found that many brain regions show a suppressed response for stimuli adjacent in feature space—and slightly varied in visual appearance—to those stimuli evoking strong neural responses. This observation indicates a form of a high-level “local inhibition”—a phenomenon often seen for simpler features encoded in earlier visual areas. Finally, a visual inspection of the stimulus images corresponding to the spatial selectivity centers of positive responses offers some intuition regarding what higher-level visual properties—for example, holistic object shape, part shape, and surface texture—are encoded in these specific areas of the brain.

Critically, an examination of the distribution of cortical responses for both visual feature spaces indicates repeating patterns across subjects and ROIs. In particular, for both the SIFT and Fribble spaces, a subset of searches show that stimuli eliciting extreme high or low responses cluster together, while stimuli eliciting responses more in the middle are spread further from cluster centers. This pattern of slightly differing stimuli producing extremely different neural responses is consistent with known visual coding principles within earlier stages of the ventral pathway. At the same time, this observation is not universal—within both the SIFT and Fribble spaces, some searches produce cortical response maxima that are distributed broadly across a given feature space, rather than concentrated in one location.

### 4.1. Proximity of differential responses

The proximity of stimuli evoking ROI responses of opposite extremes can be seen in the scatter plots in Figures 9, 11,^5^. Similar structure is apparent in the sorted stimulus images illustrated as the red figures in Figure 5D^6^. As mentioned earlier, these findings are consistent with the principle of local inhibition often observed within earlier processing stages of the visual system. For example, Hubel and Wiesel (1968) observed spatially adjacent “on” and “off” edge regions in visual stimuli exciting or inhibiting, respectively, the spiking of neurons in mammialian V1. In modern hierarchical models of the primate visual system, the first stage of processing is often held to reflect such early findings as realized as a series of Gabor filters (Serre et al., 2007; Kay et al., 2008). Even earlier within the visual system, prior to cortical coding, retinal ganglion cells are similarly known to have receptive fields characterized by concentric “on” and “off” rings in the image plane of any given stimulus (Rodiek and Stone, 1965). More broadly, multiple stages of alternating patterns of excitation and suppression are consistent with principles of successful neural coding models, in which lateral inhibition of representational units “located” adjacent to or nearby one another are found to be advantageous to a variety of visual tasks (Rolls and Milward, 2000; Jarrett et al., 2009). The sort of local competition observed in our study—that is, in alternative feature spaces—is conceptually plausible based on such models. Our findings indicate that local inhibition does indeed seem to occur in the complex representational spaces employed at more advanced stages of cortical visual object perception.

### 4.2. Visual intuitions about feature selectivity

Analysis of cortical activities over visual space provides further understanding of the presence of one or more selectivities for a given brain region and the presence of local inhibition within the defined visual space. However, intuition about the nature of preferred stimuli, and their underlying visual properties, is perhaps better obtained through visual inspection of those stimuli frequently visited by each search and evoking extreme cortical responses. For many real-world objects searches, it was not possible to identify unifying visual patterns of preferred stimuli. For some searches we did observe potentially consistent selected shape and surface properties. In particular, for Fribble object searches, executed in constrained visual spaces, unifying visual patterns for stimuli producing high cortical activity largely were holistic Fribble shapes. At the same time, there were no clear patterns across subjects regarding the preferred types of holistic shapes (which are dependent upon the shapes of the three component appendages of each Fribble class).

For both real-world and Fribble objects searches, visual inspection of the ordering of stimuli by ROI response, that is, as shown in Figures 5, 9, fails to yield any specific insights. *A priori*, we would expect shape properties to smoothly transition as measured responses decreased. That we did not observe this transition may stem from the fact that our measurements reflect a mix of multiple coding units or noise in our fMRI data (despite averaging). For real-world objects, note that the construction of our four-dimensional search space using MDS may also limit our ability to detect the fine-grained organization of the stimuli, yet maintain the broader similarity groupings of these same images. At the same time—in light of our finding of evidence for local inhibition—we might alternatively expect that that similar-looking stimuli would appear at opposite ends of the line of sorted stimuli. Interestingly, such a visual disconnect between top-ranked stimuli for single ventral pathway neurons was observed by Woloszyn and Sheinberg (2012).

More broadly, frequently visited stimuli clustered together in SIFT space—evoking both extreme high and low responses, consistent with the observations above—can be united by coarse shape (e.g., width in Figures 5D, 7C), surface properties (e.g., brightness in Figure 5D or texture for S1 class 2 in Table 1), and fine internal contours (e.g., sharp-edged holes for S5 class 2 in Table 1). This observed selectivity for shapes is consistent with the findings of Yamane et al. (2008) and Hung et al. (2012), who successfully identified two- and three-dimensional contour preferences for neurons in V4 and IT using uniform-gray blob stimuli. Unlike these prior studies, our work employs real-world stimuli and thus identifies classes of preferred shapes likely to be encountered in normal life experience. Observed selectivity for surface properties is a more novel finding, though Tanaka et al. (1991) observed such selectivities in primate IT neurons in the context of perception of object drawings. While some searches provided insights about cortically relevant visual properties, many searches performed for real-world objects revealed no clear patterns among stimuli evoking extreme cortical region responses, clustered together in SIFT-based space. This lack of clear patterns likely reflects the difficulty of capturing the diversity of real-world visual properties in a four dimensional space, as discussed in Section 3.5.

We also note that changes in the cortical representation of the stimuli due to repeated exposures across the three study sessions may have made interpretation of our results more difficult across our entire study. However, arguing against this possibility, our observation of stronger search performance for subjects viewing Fribble stimuli—novel images with significant similarity in appearance within each class (thereby increasing the likelihood of overlap in the neural representations of individual stimuli)—suggests that potential adaptation or learning effects did not constitute a significant problem.

### 4.3. Selectivity to visual parts

Fribble objects, and corresponding “Fribble spaces,” were more controlled in their span of visual properties than were the real-world stimuli. Frequently visited stimuli in each Fribble space can cluster around a three-dimensional coordinate. Each dimension corresponds to variations of a single component shape morphed between two options, such as a star/circle head or flat/curved feet, as in Figure 4A. Thus, clustering around a point indicates slight variations on three component shapes, with focus around a fixed holistic shape. However, across subjects, there was no clear pattern of preferred holistic Fribble shapes, nor of preferred shapes for any of the three varying component “appendages.” For some searches, frequently visited Fribble stimuli evoking strong cortical responses varied along one axis or two axes while staying constant on the remaining one(s). Depending on the brain region being interrogated, one to three component shapes were able to account for such selectivity.

In interpreting this result, we note that it is possible that the appendage-based construction and variations of Fribble stimuli may have biased subjects to rely on perceptual strategies focused on object parts. Nonetheless, observations on cortical responses in these subjects may be supported by evidence for parts-based neural representations observed in subjects viewing less-structured real-world objects—for example, ROIs selective for rectangular statue bases or for bag handles in Table 1. One possibility is that more local selectivity for parts of an object, rather than the whole, may be associated with cortical areas particularly earlier in the ventral pathway—an organization that would be consistent with the focus of early and intermediate stages of vision on spatially-distinct parts of a viewed image, pooled together over increasingly broader parts of the image at higher stages of vision (Riesenhuber and Poggio, 1999).

## 5. Conclusions

Our study is one of the first to address head on the challenge of identifying intermediate-level feature representation in human ventral cortex. That is, although there is a great deal known about early visual coding and increasing knowledge regarding high-level visual representation (Huth et al., 2012), the field has been relatively silent (with the exception of Tanaka, 1996, Yamane et al., 2008, Hung et al., 2012) on how simple edge-like features are combined to encode more complex features such as parts, textures, and complex shapes. Here we explored this question in two ways. First, by advancing the application of a novel research methodology—real-time methods for rapidly measuring and processing the BOLD signal on a trial-by-trial basis. Second, by introducing a new research approach as applied to human neuroimaging—search methods for efficiently seeking the image or images that are most effective in driving specific brain regions. Although our overall findings are somewhat mixed regarding what we have learned about intermediate-level neural coding, we observed sufficient consistency—in particular, with respect to apparent high-level local inhibition—to suggest that as these methods mature, they offer a promising new direction for exploring the fine-grained neural representation of visual stimuli within the human brain.

### Conflict of interest statement

The authors declare that the research was conducted in the absence of any commercial or financial relationships that could be construed as a potential conflict of interest.

## Footnotes

^1^The measured response of each voxel for each stimulus repetition consisted of five data samples starting 2 s after stimulus onset, corresponding to the 10 s between stimuli. Each five-sample response was consolidated into a single value—the average of the middle three samples of the response (Just et al., 2010)—intended to estimate the peak response.

^2^For the interpretation of real-world objects results, grouping was done by visual inspection of a single linkage dendrogram constructed in the four-dimensional SIFT-based space.

^3^Similarity in appearance is not well-defined, as explored by our past work in Leeds et al. (2013). Generally, we limit our similarity judgements to identification of identical pictures, e.g., in Figure 5. Here, we occasionally use more rough intuition.

^4^For the interpretation of Fribble results, grouping was done by visual inspection of the three-dimensional scatter plots, e.g., Figure 9A.

^5^Scatter plot examples only are given here in Fribble spaces as they are more easily evaluated visually in one two-dimensional plot.

^6^Stimulus examples only are given here for SIFT searches as similarities of the real-world object stimulus set are easier to see than they are for Fribbles that all look predominantly similar within a given class to the uninitiated reader.
