# Supplementary material for: Exploration of complex visual feature spaces for object perception
Source: Front Comput Neurosci. 2014 Sep 12;8:106. doi: 10.3389/fncom.2014.00106 (PMC4162468; doi:10.3389/fncom.2014.00106)
Supplement: Supplementary file 1 [file DataSheet1.PDF]

Frontiers in Computational Neuroscience Research Article

Supplementary Material: Exploration of complex visual feature spaces for object perception

Daniel D. Leeds [1,2,*], John A. Pyles [2,3] and Michael J. Tarr [2,3]

[1] *Fordham University, Computer and Information Science Department, Bronx, New York, USA*
[2] *Carnegie Mellon University, Center for the Neural Basis of Cognition, Pittsburgh , Pennsylvania, USA*
[3] *Carnegie Mellon University, Psychology Department, Pittsburgh , Pennsylvania, USA*

Correspondence*:
Daniel D. Leeds
Fordham University, Computer and Information Science Department, 441 East Fordham Road, Room 328A, John Mulcahy Hall, Bronx, New York, 10458, USA, dleeds@fordham.edu

1 ASSESEMENT OF SEARCH PERFORMANCE

1.1 CONVERGENCE

For a given class, convergence was computed based on the feature space locations of the visited stimuli S, and particularly the locations of stimuli visited three or more times, $\mathbf{S_{thresh}}$. The points in $\mathbf{S_{thresh}}$ were clustered into groups spanning no more than d distance in the associated space based on average linkage, where $d = 0.8$ for Fribble spaces and $d = 0.26$ for SIFT space.[1] The result of clustering was the vector $\mathbf{clusters_{S_{thresh}}}$, where each element contained the numeric cluster assignment (from 1 to N) of each point in $\mathbf{S_{thresh}}$. The distribution of cluster labels in $\mathbf{clusters_{S_{thresh}}}$ was represented as $\mathbf{p_{clust}}$, where the nth entry $p_{clust}(n)$ is the fraction of $\mathbf{clusters_{S_{thresh}}}$ entries with the cluster assignment n.

Conceptually, convergence is assessed as follows based on the distribution of points, i.e., stimuli visited at least three times:

- If all points are close together, i.e., in the same cluster, the search is considered to have converged.
- If most points are in the same cluster — or in one of a couple dominant clusters — and there are a "small number" of outliers in other clusters, the search is considered to have converged sufficiently.
- If points are spread widely across the space, each with its own cluser, there is no convergence.

Set as an equation, the convergence metric is

$$metric(\mathbf{S}) = ||\mathbf{p_{clust}}||_2 - .1||\mathbf{p_{clust}}||_0 \tag{1}$$

where $||\mathbf{p_{clust}}||_2 = \sqrt{p_{clust}(1)^2 + \cdots + p_{clust}(N)^2}$ and $||\mathbf{p_{clust}}||_0$ is the number of non-zero entries of $\mathbf{p_{clust}}$. The metric awards higher values when $\mathbf{p_{clust}}$ element entries are high (most points are in a small number of clusters) and the number of non-zero entries is small (there are few clusters in total).

[1] The distance thresholds were chosen based on empirical observations of clusterings across regions and subjects in each space.

Eqn. 1 pursues a strategy related to that of the elastic net, in which $\ell 2$ and $\ell 1$ norms are added to award a vector that contains a small number of non-zero entries, all of which have small values **Zou and Hastie** (2005).

1.2 CONSISTENCY

The metric for determining consistency of results across search sessions was a slight modification of the convergence metric. The locations of the stimuli visited three or more times in the first and second searches were stored in $\mathbf{S^1_{thresh}}$ and $\mathbf{S^2_{thresh}}$, respectively. The two groups were concatenated into $\mathbf{S^{both}_{thresh}}$, taking note which entries came from the first and second searches. Clustering was performed as above and labels were assigned into the variable $\mathbf{clusters_{Sboththresh}}$. The distribution of cluster labels was represented as probabilities $\mathbf{p_{clustBoth}}$.

To measure consistency, the final metric in Eqn. 1 was applied only to entries of $\mathbf{p_{clustBoth}}$ for which elements of $\mathbf{S^1_{thresh}}$ **and** $\mathbf{S^2_{thresh}}$ were present

$$metric(\mathbf{S^{both}}) = ||\mathbf{p_{clustBoth}}(i \in \mathbf{B})||_2 - .1||\mathbf{p_{clustBoth}}(i \in \mathbf{B})||_0 \quad (2)$$

where $\mathbf{B}$ is the set of indices i such that cluster i contains at least one point from $\mathbf{S^1_{thresh}}$ and from $\mathbf{S^2_{thresh}}$. The metric awards the highest values for convergence if there is one single cluster across search sessions. A spread of points across the whole search space visited consistently between sessions would return a lower value. Complete inconsistency would leave no $\mathbf{p_{clustBoth}}$ entries to be added, returning the minimum value of 0.

1.3 TESTING AGAINST CHANCE

As the convergence and consistency metrics above are not well established, it is not clear what values should be considered sufficiently high to indicate desirable search performance and what values would arise by chance. A variant of the permutation test is used to assess the metric results. The null hypothesis is that the convergence or consistency measure computed for a given search or pair of searches, based on clustering of the k stimuli visited three or more times during the search(es), would be equally likely to be found if the measure were based on clustering of a **random** set of k stimuli; this random set is chosen from the stimuli visited **one** or more times during the same search(es). The group of stimuli visited one or more times is considered a conservative estimate of all stimuli that could have been emphasized by the search algorithm through frequent visits. In the permutation test, the designation “displayed three or more times” is randomly reassigned among the larger set of stimuli displayed one or more times to determine if a random set of stimuli would be considered similarly convergent or consistent as the set of stimuli frequently visited in my study. More specifically, indices are assigned to all points visited in search 1 and search 2, S^1 and S^2, respectively, the indices and recorded number of visits are randomly permuted, and $metric(\mathbf{S^1})$, $metric(\mathbf{S^2})$ and $metric(\mathbf{S^{both}})$ are computed based on the locations randomly assigned to each “frequently-visited point.” For each subject and each search, this process is repeated 500 times, the mean and standard deviation are computed, and the Z score for the original search result metrics are calculated. Based on visual inspection, searches with $z \geq 1.8$ are considered to mark notably non-random convergence or consistency.

REFERENCES

Zou, H. and Hastie, T. (2005), Regularization and variable selection via the elastic net, *Journal of the Royal Statistical Society, Series B*, 67, 301–320
